# Predicting subjective refraction with dynamic retinal image quality analysis

**DOI:** 10.1038/s41598-022-07786-0

**Published:** 2022-03-08

**Authors:** Andrea Gil, Carlos S. Hernández, Ahhyun Stephanie Nam, Varshini Varadaraj, Nicholas J. Durr, Daryl Lim, Shivang R. Dave, Eduardo Lage

**Affiliations:** 1 Department of Electronics and Communications Technology, Universidad Autónoma de Madrid, Madrid, Spain; 2 Instituto de Investigación Sanitaria Fundación Jiménez Díaz, Madrid, Spain; 3 PlenOptika, Inc., Boston, MA USA; 4 Wilmer Eye Institute, Johns Hopkins University School of Medicine, Baltimore, MD USA; 5 Department of Biomedical Engineering, Johns Hopkins University, Baltimore, MD USA

**Keywords:** Engineering, Biomedical engineering, Public health

## Abstract

The aim of this work is to evaluate the performance of a novel algorithm that combines dynamic wavefront aberrometry data and descriptors of the retinal image quality from objective autorefractor measurements to predict subjective refraction. We conducted a retrospective study of the prediction accuracy and precision of the novel algorithm compared to standard search-based retinal image quality optimization algorithms. Dynamic measurements from 34 adult patients were taken with a handheld wavefront autorefractor and static data was obtained with a high-end desktop wavefront aberrometer. The search-based algorithms did not significantly improve the results of the desktop system, while the dynamic approach was able to simultaneously reduce the standard deviation (up to a 15% for reduction of spherical equivalent power) and the mean bias error of the predictions (up to 80% reduction of spherical equivalent power) for the handheld aberrometer. These results suggest that dynamic retinal image analysis can substantially improve the accuracy and precision of the portable wavefront autorefractor relative to subjective refraction.

## Introduction

The determination of an eyeglass prescription is a highly skilled task requiring the optimization of the visual perception of a subject. The optimization procedure itself can be highly variable and may be influenced by factors other than just refractive errors such as physiological fluctuations in the patient’s eye, the ability of the patient to distinguish small deviations in refractive power, or neural compensation^1^. To account for these sources of variability, initial objective measures with autorefractors are further refined iteratively by subjective refraction, the clinical gold standard for refractive error correction. The most recent report by The Lancet Global Health Commission estimates that more than 90% of people with vision impairment live in low-income and middle-income countries and have a preventable or treatable cause^2^. Thus, the ability to obtain affordable autorefraction measurements in the hands of a deskilled community health worker outside of clinical settings, has been identified as a fundamental technological need^3–5^.

The automatic determination of refractive error is a difficult problem in part due to several dynamic processes with the human visual system. Besides the natural accommodation mechanism, used to focus vision at different distances, it is well known that the power of the crystalline lens undergoes rapid and continuous changes known as microfluctuations^6^ that play an important role in ocular optics. Furthermore, the tear film and its stability over time are also known to influence the optical quality of the eye by affecting the amount of high-order aberrations (HOAs) and visual function^7–9^. Finally, depth of focus, defined as the variation in defocus, which can be tolerated by the eye without causing any objectionable change in sharpness of the retinal image, is also known to vary with neural factors, viewing conditions, and pupil size^10^. Given the dynamic nature of these factors, it is reasonable to hypothesize that refraction should consider the dynamic nature of vision to determine an optimal correction. However, the vast majority of clinical autorefraction devices use static measurements to determine the optical power of the eye^11–13^, intrinsically assuming that the quality of vision is stable between eye blinks. Several recent studies indicate that the agreement between objective and subjective refraction techniques is, in general, very good for most subjects^14,15^. However, in all the studies, there remains a non-neglectable percentage of outliers (e.g., ~ 15–60% depending on the autorefractor used and characteristics of the study population) in which clinically significant differences (> ± 0.5 D spherical equivalent difference between autorefraction and subjective refraction) have been observed. Apart from the dynamic processes already mentioned, these discrepancies have been associated with other factors including pathologies such as diabetes^16^, eye conditions such as keratoconus^17^, neural adaptation to refractive correction^1,18^ or high-order aberrations and their variations with pupil size^19^ amongst others.

Wavefront aberrometry is considered the most comprehensive objective refraction technology because it provides a detailed map of the ocular aberrations including low- (defocus and astigmatism) and high-order components. Despite the fact that the high-order aberrations cannot be effectively corrected by conventional eyeglasses, several attempts to compute the subjective refraction of a patient from objective wavefront aberrations measurements have been investigated over the last 20 years^20–24^. Furthermore, it has been demonstrated that wavefront information can be analyzed to estimate objective quality metrics that describe the optical and perceptual image quality of a subject^21,24^. Those image quality metrics (IQM), which can be based on a variety of optical^25^, neural^24^, or imaging-related^26,27^ parameters, have been used to optimize the agreement between objective and subjective refraction with different degrees of success^21,22,25,27^. When using these metrics, the refraction optimization process consists of a search in a synthetically generated 3-dimensional space to find sphere, cylinder, and axis values of a correcting lens that optimizes a certain IQM when applied to a static wavefront aberration measurement of a subject. Although this approach has shown promising results, it is a computationally intensive method because it requires evaluating hundreds to thousands of possible corrections^28^.

In this work we investigate the variations of the optical and perceptual image quality of human subjects by determining retinal IQMs from dynamic wavefront aberrometry measurements. Furthermore, we propose a novel algorithm capable of using the dynamic aberrometry information and evaluate its ability to simultaneously improve the precision and accuracy of autorefraction compared to subjective refraction.

## Materials and methods

### Patient population

Adult individuals that were free of significant ocular and systemic pathology were recruited at Johns Hopkins University School of Medicine Green Spring Station between 2017–2018^29^. Exclusion criteria were: (1) use of systemic or ocular drugs that may affect vision, and (2) history of surgery or eye disease other than strabismus. This study was approved by the Institutional Review Board at Johns Hopkins University and was adhered to the tenets of the Declaration of Helsinki. Informed consent was obtained from all participants.

### Equipment

The reference instrument in this study was the VISX WaveScan (“WS,” Software V 3.68, Visx, Inc., Santa Clara, CA, USA) desktop wavefront aberrometer. This device uses Hartmann-Shack technology to capture high-quality static images which allow measuring refractive errors and wavefront aberrations of the eye. The measurements with this device are recommended without pupil dilation and the device has been widely used for almost 20 years primarily to plan custom LASIK surgery^30–32^.

QuickSee (“QS,” PlenOptika, Inc., USA) is a handheld wavefront autorefractor based on Hartmann-Shack technology which combines a binocular open-view design and dynamic wavefront measurements (frame rate: 8 frames/second/eye). It is intended to be used as a portable autorefractor for clinical practice and vision screenings and has been reported to provide accurate measurements under a variety of ambient lighting conditions^3,33–35^. In contrast to the high-end WaveScan system, due to its portable form factor and lower cost, QS captures raw images that are of lower quality than WS in terms of alignment, signal-to-noise, and spatial resolution. In order to improve the accuracy of its autorefraction despite the lower quality of raw data, QS captures a large sequence of images and utilizes advanced algorithms that leverage this dynamic content^33^.

### Data acquisition protocol

Subjective refraction was recorded for each subject enrolled as the prescription within 6 months of the visit to the eye clinic. In all cases the refraction was performed at JHU Medical facilities following standard clinical guidelines. During a single session, patients were measured with both refraction devices under non-cycloplegic and consistent lighting conditions in a room with no windows and lights turned off. Three sequential monocular measurements of each eye were obtained for all subjects with the WaveScan, which measured the pupil size and Zernike coefficients up to the 4th order. Three binocular measurements were also taken with the QuickSee for each patient. The QuickSee recorded raw Shack-Hartmann spot patterns for each eye for 10 s, together with the corresponding pupil size, and Zernike coefficients^36^ up to the 4th order for each spot pattern. In all cases, we used the manufacturer recommended settings for the devices and aberrometry data (pupil size, Zernike coefficients) as originally provided by each device.

For analysis, the static aberration data from WaveScan was used together with standard search-based IQM optimization procedures^28^. Dynamic data from QuickSee were used to evaluate the performance of the dynamic retinal image quality optimization algorithm proposed in this work. Reference values used for comparison of each independent approach were WaveScan refraction (WaveScan AR, static approach), and QuickSee refraction (QuickSee AR, dynamic approach), without applying any optimization. In all cases, optimized and non-optimized results were compared against subjective refraction of each patient.

### Retinal image quality metrics (IQM)

The retinal image quality metrics used in this study are a subset of previously published metrics in the literature^21,28,37^. They are based on the distribution of light and the optical quality of the point spread function (PSF), which is defined as the response of an eye to a point source. In all cases, the PSF of the eyes was calculated for each measurement using Zernike coefficients up to the 4th order. Programs for computing the PSFs and IQM were written in Matlab R2020b (The MathWorks, Inc.). A summary description and definition of these IQM can be found in Table 1.

**Table 1:** Retinal image quality metrics used in the optimization methods.

Abbreviation	IQM	Description
SR	Strehl ratio	It is defined as the ratio of the maximum peak of the PSF of an optical system over that of a diffraction-limited optical system (PSF_DL_) with the same pupil size^28,37^: $SR= \frac{max(PSF)}{max({PSF}_{DL})}$
FWHM	Full width at half maximum	It is defined as the full width at half maximum of all the cross sections of the PSF of an optical system^37^
Entropy	Entropy	It is a measure of the spatial variance of the PSF that analyses how the energy is distributed in the image^21^: $\mathrm{Entropy}= -\sum_{x, y}PSF\left(x,y\right)\times \mathrm{log}PSF(x,y)$
IV	Intensity variance	It is calculated as the average value of squared PSF minus the average PSF squared^21^: $IV= \overline{PS{F }^{2}}-PS{F}^{2}$
STD	Standard deviation of intensity values in the PSF	It measures the variability of intensities at various points in the PSF^28^: $STD= \frac{{\left[\int {\left(PSF\left(x,y\right) - \overline{PSF }\right)}^{2}dx dy\right]}^{0.5}}{{\left[\int {\left({PSF}_{DL}\left(x,y\right) - \overline{{PSF }_{DL}}\right)}^{2}dx dy\right]}^{0.5}}$
NS	Neural sharpness	The PSF is weighted by a neural weighting-function (bivariate-Gaussian, g), integrated and normalized by the corresponding value for a diffraction-limited PSF^28^: $NS= \frac{\int PSF\left(x,y\right)\cdot g\left(x,y\right)dx dy}{\int {PSF}_{DL}\left(x,y\right)\cdot g\left(x,y\right)dx dy}$
VSX	Visual Strehl ratio computed in the spatial domain	The PSF is weighted by a bivariate neural weighting-function (inverse Fourier transform of the neural contrast sensitivity function, C), integrated and normalized by the corresponding value for a diffraction-limited PSF^28^: $VSX= \frac{\int PSF\left(x,y\right)\cdot C\left(x,y\right)dx dy}{\int {PSF}_{DL}\left(x,y\right)\cdot C\left(x,y\right)dx dy}$
VSMTF	Visual Strehl ratio computed in frequency domain	The modulation transfer function (MTF, the absolute value of the Fourier transform of PSF) is weighted by the neural contrast sensitivity function CSF_N_, integrated and normalized by the corresponding value for a diffraction-limited MTF^28^: $VSMTF = \frac{{\mathop {\iint }\nolimits_{{ - \infty }}^{{ + \infty }} CSF_{N} \left( {f_{x} ,f_{y} } \right) \cdot MTF\left( {f_{x} ,f_{y} } \right)df_{x} df_{y} }}{{\mathop {\iint }\nolimits_{{ - \infty }}^{{ + \infty }} CSF_{N} \left( {f_{x} ,f_{y} } \right) \cdot MTF_{{DL}} \left( {f_{x} ,f_{y} } \right)df_{x} df_{y} }}$

## Data analysis

### Optimization procedure

#### Static approach (on WaveScan)

The optimization procedure with static data consists of a brute-force search algorithm in which several possible corrections are computationally applied to each eye to determine which one performs better at optimizing a certain parameter of the vision of a patient represented by a IQM^21,22,25,27^. To generate the search space, ranges of spherical power of ± 1.5 D surrounding the final sphere provided by the WS desktop system were tested in 0.25 D steps. For the cylindrical power, we tested ± 1 D surrounding the final cylinder result in 0.25 D steps. For the axis, steps of 5° were used over ± 20° range. The resulting sphero-cylindrical corrections (up to 1,215 combinations per eye) were converted to low-order Zernike coefficients in the pupil plane and added to the second-order Zernike coefficients provided by the devices to estimate the residual aberrations of each possible correction. These residual wavefront errors, containing low and high order terms, were used to calculate the PSF of the corrected eye, which was finally used to determine the IQM described in Table 1. All the low-order Zernike terms used in the calculations had the corresponding chromatic correction applied.

In order to select the final correction for each IQM metric, the mean of power vectors within the best 5% metric performance was used. This was preferred, instead of choosing the prescription with the maximum or minimum score (depending on the metric), since it ensures that similar prescriptions with scores that are close to each other are incorporated into the analysis.

#### Dynamic approach (on QuickSee)

The dynamic analysis of retinal image quality attempts to account for the fact that optical aberrations (refractive errors) are continuously fluctuating due to dynamic physiological phenomena such as accommodation or tear film breakup. The magnitude of such fluctuations has been studied previously, and they contribute to the uncertainty from basing refractive error correction on a single (or few) static measurement(s). The proposed optimization procedure herein, consists of evaluating how a given IQM changes with different values of refractive error measured dynamically in order to select the refraction that best optimizes the metric. Consequently, the dynamic approach uses a search space consisting of the different refractions measured for the eye during a dynamic acquisition, instead of performing a three-dimensional search of an optimized prescription in a synthetically generated search space.

**Figure 1:**
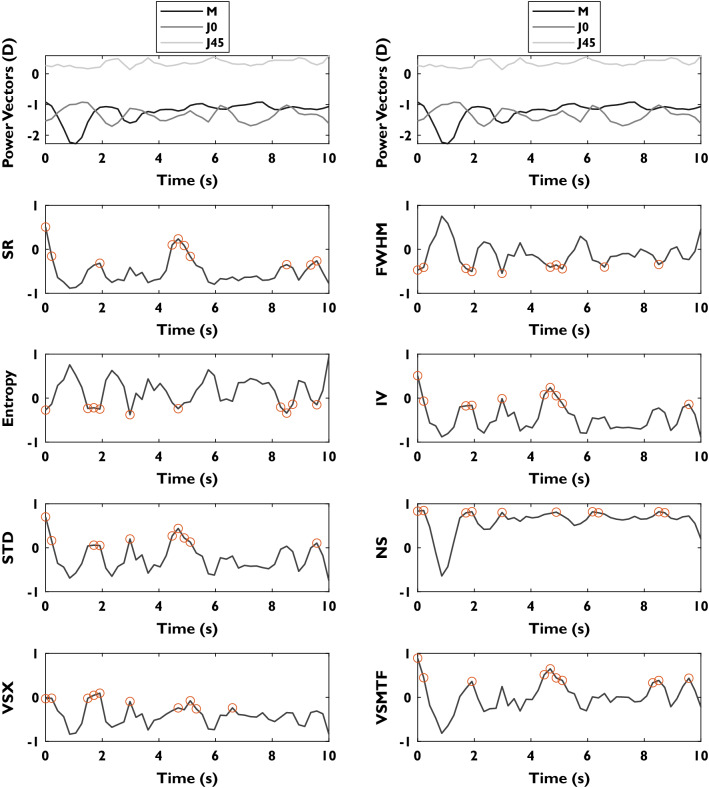
Dynamic signal plot showing refractive errors (M, J0 and J45) and the resulting IQM (normalized units) calculated during a QuickSee measurement of 10 s. This patient (69 years old) shows variability in the spherical equivalent dynamic signal during the first two seconds of the measurement. Best metric performance regions are represented as orange points in the signals.

After an initial filtering to discard empty frames due to blinks (directly labeled by the QS software), for each frame in the acquired video, the spot pattern was processed to obtain a Zernike coefficient set, which was mathematically corrected using the closest sphero-cylindrical corrections (converted to low-order Zernike coefficients in the pupil plane). Residual wavefront errors of each frame, containing low- and high-order terms, were used to calculate the PSF and IQM of the corrected eye. Since each IQM is part of a dynamic sequence, it is possible to build a dynamic signal for each metric (Fig. 1) which contains information about fluctuations in image quality during the measurement. Low-order Zernike terms used in the calculations had the corresponding chromatic correction applied. The final refraction for each IQM was obtained from the mean of power vectors within the top 20% metric performance (Fig. 1). In all cases, FWHM and Entropy are the unique metrics that should be minimized to improve corrections, while the rest must be maximized.

### Data analysis

Power vectors have been used as the sphero-cylindrical descriptors (as opposed to optometric notation) including spherical equivalent (M, Eq. 1), and the two power terms of Jackson cross-cylinder, J0 (Eq. 2) and J45 (Eq. 3).

1 $$\mathrm{M }=\mathrm{ sphere\,error}+ \frac{\mathrm{cylinder\,error}}{2}$$

2 $$\mathrm{J}0 = - \frac{\mathrm{cylinder\,error}}{2}\cdot cos(2\,axis)$$

3 $$\mathrm{J}45 = -\frac{\mathrm{cylinder\,error}}{2}\cdot sin(2\,axis)$$

Results obtained with the WS using the static approach, and the results obtained using the QuickSee in dynamic mode were compared against the gold standard of subjective refraction. Precision of each IQM was defined as twice the standard deviation of the differences between the predicted refraction and subjective refraction for M, J0, and J45; this corresponds to the 95% limits of agreement of Bland–Altman analysis. Accuracy for spherical equivalent (M) was computed as the mean bias error (MBE). Mean absolute error (MAE), agreement percentages within 0.25 D and 0.5 D, and the distribution of error for best performing metrics were also evaluated. Only data from the right eye is reported in the analysis.

## Results

Forty-one adult individuals (age 53 ± 17 years) were recruited for this study. 6 measurements for each subject (3 from each eye) were obtained for only 34 subjects (age 50 ± 18, range 19–82 years old) (Table 2) using both the desktop WaveScan (WS) system and the portable QuickSee (QS). For the remaining 7 subjects, readings could not be taken with at least one of these two devices. In this age range, average amplitude of accommodation is expected to be around 1.5 D ± 1D, although large interindividual differences can be also expected^38^. Final sample size was n_QS_ = 101, n_WS_ = 102 for the QS and WS, respectively, since 1 measurement from QS was erased accidentally. Based on manifest-refraction data, non-corrected sphere in the right eyes ranged from − 6.25 to + 3 D and the cylindrical component ranged from 0 to − 3 D. Table 2 describe the patient population classified by refractive error group.

**Table 2:** Refractive error in the right eyes of the patient population as determined by manifest refraction.

Eye condition	# Patients
**Hyperopia**	
0.5 ≤ S < 3 D	12
S ≥ 3 D	1
**Myopia**	
− 3 < S ≤ − 0.5 D	10
S ≤ − 3 D	4
**Astigmatism**	
− 1.5 < C < 0 D	28
C ≤ − 1.5 D	3
**Emmetropia**	
− 0.5 < M < 0.5 D	10

Accuracy for measuring refraction was assessed looking at the mean difference (MBE), between the subjective and the objective methods (QS or WS). These results together with values of the mean absolute error (MAE) and the 95% limits of agreement (LOA) can be found in Table 3.

**Table 3:** Mean absolute error (MAE), mean bias error (MBE) and 95% limits of agreement (LOA) achieved by different IQMs and approaches to determine power vectorsrefraction (Autorefractors, AR, vs Subjective Refraction).

	AR	SR	FWHM	Entropy	IV	STD	NS	VSX	VSMTF
**M**									
Static WaveScan n = 102									
MAE (D)	0.36	0.36	0.37	0.34	0.35	0.35	0.36	0.35	0.36
MBE (D)	0.07	0.07	0.09	0.07	0.07	0.07	0.09	0.06	0.07
95% LOA (D)	0.86	0.86	0.87	0.84	0.85	0.85	0.86	0.85	0.86
Dynamic QuickSee n = 101									
MAE (D)	0.42	0.36	0.37	0.35	0.36	0.37	0.35	0.35	0.36
MBE (D)	− 0.15	− 0.07	− 0.05	− 0.02	− 0.04	− 0.05	− 0.03	− 0.03	− 0.06
95% LOA (D)	1.14	1.04	1.04	0.98	1.03	1.04	0.96	1.02	1.03
**J0**									
Static WaveScan n = 102									
MAE (D)	0.16	0.14	0.14	0.14	0.14	0.14	0.14	0.14	0.14
MBE (D)	− 0.04	− 0.03	− 0.03	− 0.03	− 0.03	− 0.03	− 0.04	− 0.03	− 0.03
95% LOA (D)	0.41	0.38	0.38	0.37	0.38	0.38	0.39	0.38	0.37
Dynamic QuickSee n = 101									
MAE (D)	0.23	0.23	0.22	0.22	0.23	0.22	0.21	0.22	0.24
MBE (D)	− 0.01	− 0.01	0.00	0.00	− 0.01	0.00	− 0.01	− 0.01	0.00
95% LOA (D)	0.64	0.62	0.60	0.59	0.61	0.60	0.58	0.61	0.62
**J45**									
Static WaveScan n = 102									
MAE (D)	0.10	0.10	0.10	0.10	0.10	0.10	0.10	0.10	0.10
MBE (D)	− 0.02	− 0.02	− 0.03	− 0.02	− 0.02	− 0.02	− 0.03	− 0.02	− 0.02
95% LOA (D)	0.24	0.24	0.24	0.25	0.25	0.25	0.25	0.25	0.24
Dynamic QuickSee n = 101									
MAE (D)	0.14	0.13	0.12	0.13	0.13	0.13	0.12	0.13	0.13
MBE (D)	− 0.06	− 0.04	− 0.03	− 0.03	− 0.03	− 0.03	− 0.03	− 0.02	− 0.03
95% LOA (D)	0.36	0.36	0.34	0.35	0.36	0.35	0.34	0.34	0.35

All results are rounded to the second decimal.

The initial M MAEs of autorefractors were 0.36 D and 0.42 D for WaveScan AR and QuickSee AR, respectively. By optimizing the image quality metrics using the WS data and the static approach, the results in M were slightly improved or not affected in all cases except for the FWHM metric (MAE increased by 3.1%).

The same tendency was observed also for M MBE and 95% LOA with all IQM, which were demonstrated to provide small improvements over the initial results of the WS device. In general terms, the metrics with the best performance for MAE, MBE, and LOA were Entropy and VSX.

**Figure 2:**
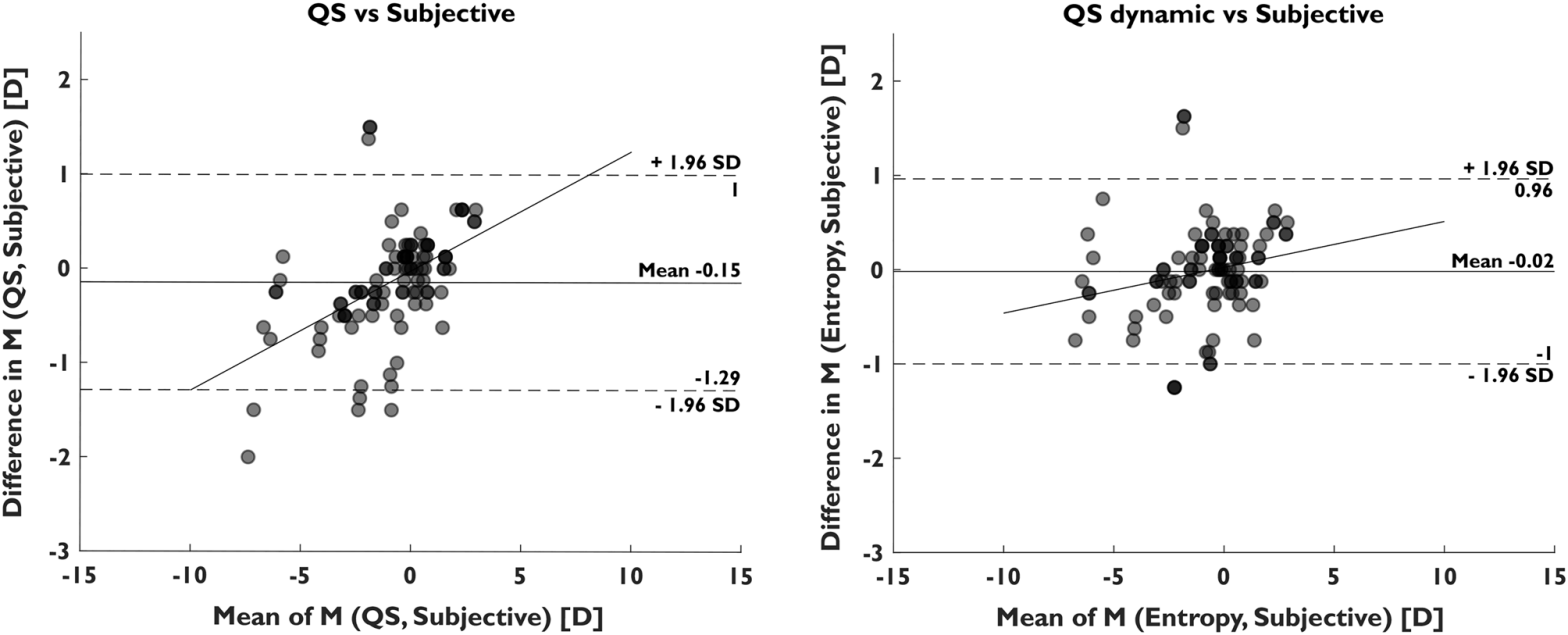
Bland–Altman plots of (left) QuickSee autorefractor measurements versus subjective refraction and (right) dynamic approach using Entropy IQM for QuickSee versus subjective refraction in the same patients (n = 101 in both cases). The line represents a linear fitting of the dot’s distribution and regions that are denser are shown darker.

The dynamic approach using QS data in contrast, consistently provided a substantial improvement in M over the initial results of the QS device, in MBE (~ 70% average reduction), moderate improvements for MAE (14.6% average reduction), and 95% limits of agreements (~ 10.6% average reduction) for all IQM evaluated. The best performing IQM in the dynamic QS approach was Entropy, which achieved a reduction in M of 17.8%, 14.1% and 87.3% in MAE, 95% LOA, and MBE, respectively. A Bland–Altman plot comparing the initial measurements of the QuickSee autorefractor and the results from the best performing metric (Entropy) against subjective refraction is shown in Fig. 2. Neural sharpness and VSX metrics performed slightly worse than Entropy, but still achieved comparable results (Table 3).

Absolute mean errors, mean bias errors and 95% limits of agreement in J0 and J45 between subjective and objective refraction were also computed (Table 3). For the static WS, mean J0 total improvement considering all metrics together is shown for all the analysis, MAE (9.3%), MBE (22.7%) and LOA (7.7%); while almost no improvements were observed for J45 (MAE, -0.9%; MBE, 5.3%, and LOA, -0.8% mean total improvement). For the dynamic QS approach, the MAE of cartesian and oblique power vectors improved (J0, 3.9%; J45, 8.5% mean total improvement), as well as the MBE (J0, 60.8%; J45, 52.9% mean total improvement) and LOA (J0, 6.4%; J45, 3.1% mean total improvement). Nevertheless, all the reported improvements are of lower magnitude than the ones for the spherical equivalent.

Table 4 shows the agreement of each power vector (M, J0, J45) within 0.25 D and 0.5 D of subjective refraction. Maximum agreement found in M within 0.25 D with each of the approaches was 51% (Static WS, IV) and 65.3% (Dynamic QS, VSMTF). The best results for WaveScan using the static approach for the 0.25 D threshold for M, was found to be IV (4% improvement), Entropy (2% improvement), and STD (2% improvement), respectively. However, higher improvements were found for the dynamic approach with QS, in which all the metrics enhanced the mean percentages below the 0.25 D threshold. NS (14% improvement) was one of the best performing metrics. Similar results were obtained for the 0.5 D threshold. Maximum agreement found in M within 0.5 D was 82.4% (Static WS, VSX), and 82.2% (Dynamic QS, Entropy).

**Table 4:** Agreement (≤ 0.25 D, ≤ 0. 5 D) in percentage (%) of each objective method (Autorefractor, AR) with subjective refraction and of each IQM with subjective refraction.

	AR	SR	FWHM	Entropy	IV	STD	NS	VSX	VSMTF
**Static WaveScan** **n = 102**									
M									
≤ 0.25 D	49	48	47.1	50	51	51	47.1	48	50
≤ 0.5 D	81.4	79.4	78.4	80.4	80.4	80.4	80.4	82.4	80.4
J0									
≤ 0.25 D	76.5	80.4	82.4	83.3	82.4	81.4	78.4	81.4	82.4
≤ 0.5 D	98	98	98	98	98	98	98	98	98
J45									
≤ 0.25 D	96.1	96.1	96.1	96.1	96.1	96.1	96.1	96.1	96.1
≤ 0.5 D	100	100	100	100	100	100	100	100	100
**Dynamic QuickSee** **n = 101**									
M									
≤ 0.25 D	56.4	63.4	61.4	61.4	63.4	61.4	64.4	63.4	65.3
≤ 0.5 D	74.3	80.2	81.2	82.2	81.2	80.2	81.2	79.2	80.2
J0									
≤ 0.25 D	69.3	70.3	71.3	70.3	70.3	70.3	73.3	72.3	69.3
≤ 0.5 D	89.1	88.1	89.1	89.1	88.1	89.1	91.1	87.1	88.1
J45									
≤ 0.25 D	84.2	91.1	92.1	94.1	92.1	93.1	92.1	94.1	91.1
≤ 0.5 D	97	97	97	97	97	97	97	97	97

All results are rounded to the first decimal.

**Figure 3:**
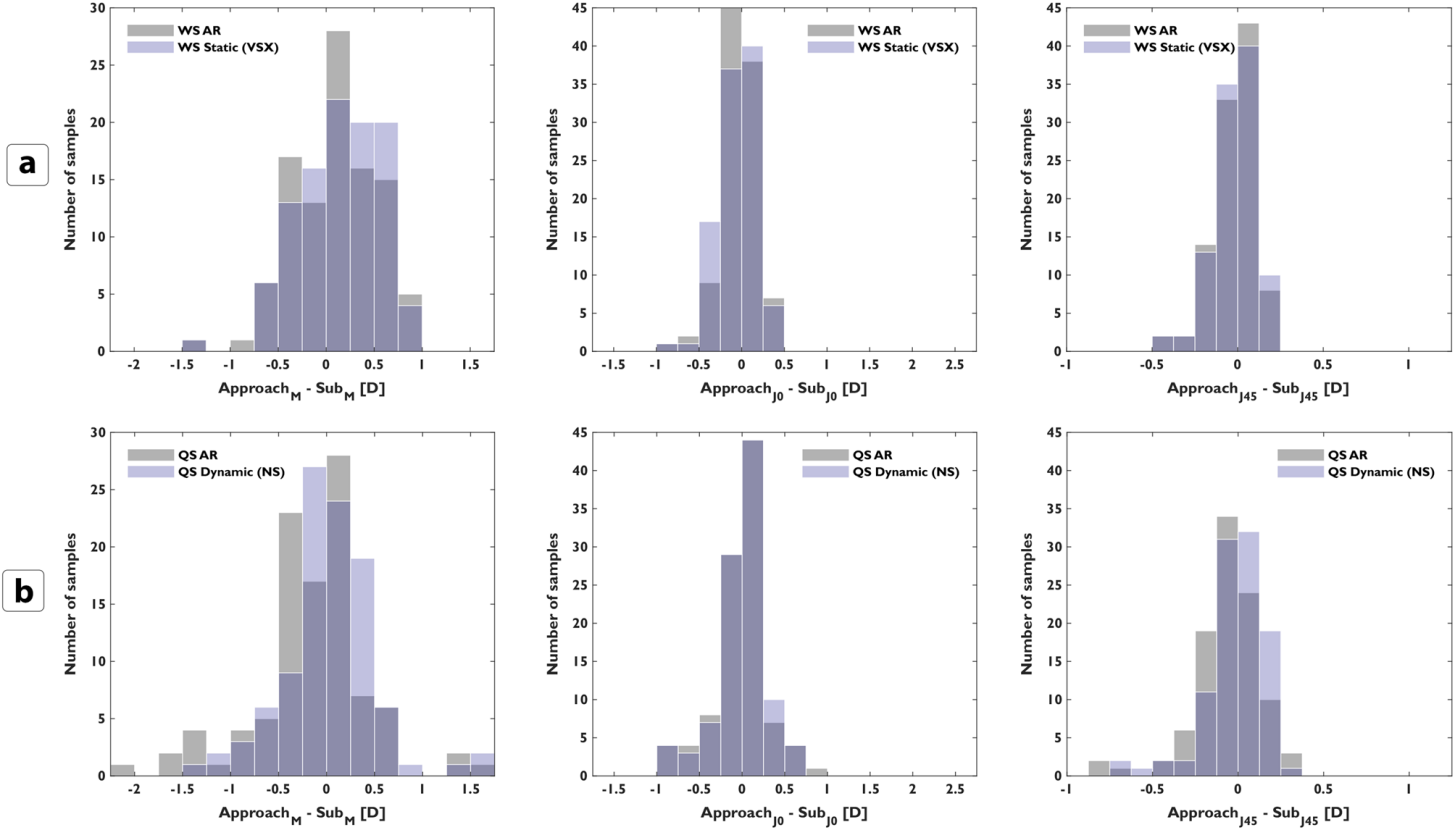
Distribution of the differences in power vectors M, J0 and J45 of each approach (static, dynamic) with respect to subjective refraction. First row (**a**) shows the differences of WaveScan autorefractor (WS AR) measurement versus the static approach (metric: VSX), and second row shows (**b**) the initial QuickSee measurement (QS AR) versus the dynamic approach (metric: NS).

Figure 3 shows the distribution of the difference in M, J0, and J45 with respect to subjective refraction before and after the application of the retinal image quality optimization for static (a) and dynamic (b) data. One of the best performing metrics was selected for each device, VSX for static WaveScan, and NS for dynamic QS.

## Discussion

Image quality metrics analyze different characteristics of light in the computed retinal image and are thus important factors when considering visual acuity. They have been widely used in search-based optimization procedures^21,27,28^. This method has been replicated in the present study using the wavefront information provided by a desktop wavefront aberrometer (static WaveScan approach). In addition, we have proposed a new procedure to predict subjective refraction that benefits from the wavefront aberrometry dynamics captured by a handheld device and retinal image quality metrics (dynamic QuickSee approach). In general, dynamic measurements have been minimally explored in the literature, but the investigations that have been performed reveal that capturing the variability of the visual process helps to understand the mechanism of vision and to achieve better visual outcomes^39–41^.

In our study, we found that the accuracy for predicting the sphero-cylindrical refraction of the 34 individuals was significantly improved for all the IQM when using the dynamic approach. The total absolute MBE for this approach was reduced from initial − 0.15 D up to − 0.02 D (Entropy), increasing the accuracy by more than 80%. Thibos et al. reported similar outcomes testing 33 metrics in statically-acquired images of 200 eyes using a laboratory Shack–Hartmann wavefront sensor system (Table 5). In that study, the mean bias provided by the least-squares fitting (-0.39 D) standard algorithm was reduced for almost all the metrics^28^. Nevertheless, the static approach in our study hardly reduces the initial bias from WaveScan (0.07 D), but the starting bias is much closer to zero than the reference study conducted by Thibos. Similar effects are observed for the precision of spherical equivalent prediction; the static approach does not considerably improve the results, while the dynamic approach reduces the lower limits of the Bland–Altman analysis for most of the metrics.

**Table 5:** Previous publications using retinal IQM for predicting subjective refraction compared to the case study.

Publication	Guirao et al. 2003^21^	Guirao et al. 2003^21^	Thibos et al. 2004^28^	Case study (static)	Case study (dynamic)
Sample Size	n = 6	n = 146	n = 200	n = 102	n = 101
Aberrometer	Laboratory Shack–Hartmann wave-front sensor system	Laboratory Shack–Hartmann wave-front sensor system	Laboratory Shack–Hartmann wave-front sensor system	WaveScan	QuickSee
Initial MAE (M)	0.5 D	–	–	0.36 D	0.42 D
Optimized MAE (M)	0.1 D (Entropy)	0.5 D (Entropy)	–	0.34 D (Entropy)	0.35 D (Entropy, VSX, NS)
Initial MBE (M)	–	–	− 0.39 D	0.07 D	− 0.15 D
Optimized MBE (M)	–	–	− 0.07 D (VSX) − 0.36 D (Entropy)	0.06 D (VSX)	− 0.02 D (Entropy)
Initial LOA (M)	–	–	0.75 D	0.86 D	1.14 D
Optimized LOA (M)	–	–	0.70 D (Entropy) 0.60 (NS)	0.84 D (Entropy)	0.96 D (NS)

A summary table with the main results of this study compared to previous publications is presented in Table 5. Best metric results are shown for the case study in terms of spherical equivalent. According to a previous study evaluating different metrics, neural sharpness metric best described the subjective impact of each patient wave aberration and Strehl ratio was found as a poor predictor^24^. This is in line with the results of our study, in which we have additionally found Entropy to be another of the best performing retinal IQM.

Guirao et al. reported an enhanced average error in M across six eyes from 0.5 D to 0.1 D (80% improvement) using five different image plane metrics. Experimental error increased to 0.4 D in sphere and 0.2 D in cylinder when a larger population was considered in that study (Table 5)^21^. Similar mean errors in spherical equivalent were found in this study 0.36 D and 0.42 D for WaveScan and QuickSee, respectively. The use of the static method did not significantly improve the results in WaveScan obtaining a reduced absolute error of 0.34 D (Entropy, WS). However, dynamic QS results were improved for all the cases, reducing the absolute error in the spherical equivalent from 0.42 D up to 0.35 D (Entropy, VSX and NS, QS). Similar improvement in M was found using a completely different method that combines artificial intelligence learning models and aberrometry to predict subjective refraction^42^.

One of the limitations of our study is that manifest refraction used to compare against autorefraction techniques and algorithms was not measured during the testing session. Instead, this value was obtained from electronic medical records within six months before the testing session. Although in all cases the standard refraction was performed at JHU Medical facilities using standard clinical guidelines with respect to testing distance and room illumination, the study is intrinsically assuming that there were no variations in subjective refraction during that 6-month period. Furthermore, no visual acuity measurements were taken with the different prescriptions (e.g., manifest refraction vs autorefraction vs IQM based refraction) which would have provided additional quantitative information about the performance of the proposed methodology.

Another notable aspect of this study is the choice of a study population with reduced accommodative amplitude (mean age 50 ± 18, expected average amplitude of accommodation 1.5 D ± 1D^38^). In this population, while micro fluctuations in accommodation remain significant, they are typically of a smaller magnitude compared to younger patients^43^. Thus, besides accommodation, increased depth of focus, due to reduced pupil size caused by aging, is expected to be another important contributor to differences between objectively and subjectively measured refraction. Unfortunately, depth of focus is difficult to measure as it is dependent on a wide range of factors including neurological and perceptual tolerance to blur^10^, or HOA, which in turn varies with tear film dynamics and pupil size^7,8^. It is thus unclear the role with which depth of focus has played in our results. That said, the considerable improvement, achieved almost independently of the IQM used, suggests that the impact of depth of focus was at least somewhat mitigated by our approach. Although it's more appropriate for a speculative study to start with an adult population, it will be important to include more patients with completely active accommodation capabilities for future studies.

In this initial investigation we have demonstrated that dynamic retinal image analysis can improve the accuracy and precision of autorefraction results relative to subjective refraction. The dynamic algorithm seems to behave as an efficient filter which select those measurements within the dynamic sequence that are more representative of the refractive error that is closest to the subjective refraction of the patient. Our results also suggest that accounting for the dynamic variations of low- and high-order aberrations rather than iterating only over different low-order corrections helps to provide a better estimate of the refractive error.
